# Mixed-gender small-sided recreational team handball games in middle-aged and elderly are physiologically more demanding for women than men

**DOI:** 10.1371/journal.pone.0286008

**Published:** 2023-06-23

**Authors:** Ivone Carneiro, Peter Krustrup, Carlo Castagna, Rita Pereira, Susana Póvoas

**Affiliations:** 1 Research Center in Sports Sciences, Health Sciences and Human Development, CIDESD, University of Maia, Maia, Portugal; 2 Department of Sports Science and Clinical Biomechanics, SDU Sport and Health Sciences Cluster (SHSC), University of Southern Denmark, Odense, Denmark; 3 Danish Institute for Advanced Study (DIAS), University of Southern Denmark, Odense, Denmark; 4 Sport and Health Sciences, University of Exeter, Exeter, United Kingdom; 5 Shanghai University of Sport (SUS), Shanghai, China; 6 Department of Biomolecular Sciences, School of Exercise and Health Sciences, Carlo Bo Urbino University, Urbino, Italy; 7 Laboratory of Metabolism and Exercise (LaMetEx), Research Centre in Physical Activity, Health and Leisure (CIAFEL), Faculty of Sport, University of Porto, Porto, Portugal; 8 University of Maia, Maia, Portugal; Università degli Studi di Milano: Universita degli Studi di Milano, ITALY

## Abstract

This study examined the physical and physiological demands and perceived experience of a multicomponent exercise mode, recreational team handball (TH), for middle-aged/elderly men and women, played as same- vs. mixed-gender 6v6 game formats. Matches’ heart rate (HR), blood lactate (BL), perceived experience, activity profile, player load and accelerometer variables were assessed. Forty-one participants, with at least 12 weeks of experience with recreational TH (22 men; 69±4 years, 19 women; 66±6 years), performed 2 same- and 2 mixed-gender matches on an indoor 40x20 m TH court. A game format-by-gender interaction was observed for mean HR (%HR_max_), time spent ˃80 and ˃90%HR_max_, respiratory rating of perceived exertion and for several of the external load variables (*p≤*0.05). During mixed-gender matches, time spent ˃80 and ˃90%HR_max_, was higher for women vs. men (*p≤*0.017). During same- and mixed-gender matches, BL was lower for women than men (*p≤*0.015). Time spent ˃90%HR_max_ was lower for women (*p =* 0.036), whereas time spent ˃80%HR_max_ was higher for men during same- vs. mixed-gender matches (*p =* 0.034). The frequency, %total match time and distance covered with high-demanding movements were higher for men during same-gender than during mixed-gender matches (*p≤*0.036), and higher for men vs. women in same- and mixed-gender matches (*p≤*0.046). The frequency of high-intensity actions, accelerations, time spent in the higher player load zones and total accumulated player load, were higher for men vs. women during same- and mixed-gender matches (*p≤*0.044). Fun levels were very high (9.1–9.3 AU, 0–10). Mixed-gender small-sided recreational TH games are physiologically more demanding for middle-aged/elderly women compared to men. Men showed higher cardiovascular and activity profile demands when playing same-gender matches, which was opposite to women. Nevertheless, TH is a high-intensity and motivating exercise mode for both genders, regardless the gender game format, meaning that exercise interventions may use same- and mixed-gender matches to promote participants’ health.

## Introduction

Multicomponent exercise interventions (i.e. a combination of resistance, endurance, and balance training) have shown to be the best strategy to improve the overall health status of frail elderly individuals [1]. Recreational team handball (TH) is a multicomponent exercise, as it requires high levels of maximal strength and muscle power to sustain the forceful muscle contractions requested during TH specific movements, a high aerobic and anaerobic turnover to cope with the intermittent high-intensity nature of the game and specific agility and balance to respond to frequent changes of direction, speed and actions [2,3].

Played as formal (i.e., 7v7) or small-sided games (3v3 to 6v6), TH has shown to improve cardiometabolic health and physical fitness of different populations with different levels of fitness, and with or without experience with the sport [4–7]. Additionally, it has proved to induce positive musculoskeletal adaptations in young adult men [5] and women [8] and in postmenopausal women [9].

Recreational TH activity profile has only been described in detail for adult/middle-aged formerly trained men [3], male college students, [10] and unexperienced older men [11]. For these participant groups, the average distance covered was 3–5 km during 40–60 min matches and 40–54 specific TH high-intensity game actions were performed, which may provide a combined positive impact on cardiovascular, metabolic, and musculoskeletal health. These physical demands imposed by recreational TH practice alongside with the high physiological stress [mean heart rates (HR): 77–85% of maximal HR (HR_max_), time spent ˃90%HR_max_: 4–22% of total match time and average blood lactate (BL): 3.6–4.4 mmol·l^-1^] have been suggested as the main reasons for the broad-spectrum health benefits observed in the studied populations [3,10,11].

The studies reporting the health and physical fitness effects of recreational TH interventions have only been organized as gender-specific exercise programs. The possibility of having mixed-gender groups in recreational TH interventions, which mainly use match-playing as training tool, is of practical advantage in a community setting where classes frequently include participants of both genders.

Although time-course aging-related changes differ in men and women, it is known that the cellular and molecular mechanisms of aging are initially better maintained in women. However, after menopause, women seem to catch up and, in several parameters, reach the same levels of aging as men [12]. Nevertheless, elderly men are still stronger and faster than women, which is probably related to higher testosterone levels resulting in higher muscle mass in men [12]. Moreover, in young adult men and women, sex differences in fatigue and ability to recover during high-intensity training are shown even when matching for exercise parameters such as maximal oxygen uptake (VO_2max_) [13,14]. Despite male superior absolute performance due to genetic differences [15,16], questioning the valence of proposing mixed-gender game formats in recreational team sports-based exercise interventions, similar cardiovascular, metabolic and bone health improvements were shown both for men and women, after 16 weeks of mixed-gender recreational football training [17]. Nonetheless, to the best of our knowledge, the physiological demands and activity profile of same- vs. mixed-gender large and small-sided games have only been analysed for children and adolescents during football practice and in a school setting [18,19]. HR response and perceived experience (namely the perceived effort and fun levels) showed a significantly lower mean value for girls when playing mixed with boys than when playing same-gender matches. No differences were reported in the game demands for boys when playing mixed- or same-gender matches [18]. However, boys perceived less fun when playing with girls than when playing within the same gender [18]. Interestingly, no significant differences were found in HR response between the genders for 8- to 9-year-old schoolchildren during small-sided team sports [19].

Therefore, understanding whether the physical and physiological demands of middle-aged and elderly men and women playing same- vs. mixed-gender recreational TH game formats differs is of utmost importance. Moreover, since motivation and enjoyment are key factors for long-term adherence to this type of exercise programmes [20], it is also important to ascertain the perceived experience during these different game formats.

Thus, the aim of this study was to analyse the physiological response, the activity profile and the perceived experience of middle-aged and elderly men and women playing same- vs. mixed-gender recreational TH game formats. We hypothesized that the demands for men would be higher when playing same- vs. mixed-game formats, while the opposite would occur for women.

## Materials and methods

### Participants

Forty-one participants (22 men and 19 women) were invited to participate in this study. Descriptive characteristics of the participants are presented in Table 1. Men’s stature, body mass, and distance covered in the Yo-Yo intermittent endurance level 1 test (YYIE1) were higher than women’s (*p≤*0.038), while body mass index (BMI), fat mass values and time experience with recreational TH, were higher for women than men (*p˂*0.001). No differences were shown between the genders for chronological age. Inclusion criteria were: male and female participants aged over 50 years, that were at the moment involved in a recreational TH-based training programme for at least the last 12 weeks, with medical clearance to perform this type of exercise program.

**Table 1:** Chronological age, stature, body composition, aerobic performance and recreational team handball experience (data are presented as mean ± SD (range)) of the men (n = 22) and women (n = 19) that played the recreational team handball matches.

Variable	Men (n = 22)	Women (n = 19)
Age (years)	69±4 (63–76)	66±6 (56–77)
Stature (cm)	168±5 (159–177)	153±4 (148–161)^*^
Body mass (kg)	74.8±8.6 (51–85)	64.7±9.0 (52–79)^*^
BMI	26.3±2.6 (20–30)	27.6±3.8 (21–34)^*^
Fat mass (%)	25.3±4.8 (15–36)	37.3±4.7 (31–44)^*^
YYIE1 (m)	754±439 (320–1560)	395±158 (200–600)^*^
Recreational TH experience (months)	17±7 (4–24)	28±12 (3–36)^*^

BMI–Body mass index; TH–Team handball; YYIE1 –Yo-Yo intermittent endurance level 1 test.

^*^Significantly different from men (*p≤*0.038).

All the participants were informed about the study purposes, risks and benefits and signed a written informed consent according to the Declaration of Helsinki. Ethical approval was provided by the local Institutional Review Board (CEFADE 19 2019).

### Experimental design

The participants were evaluated for anthropometric variables, body composition and YYIE1 performance, in the week before the data collection. All the participants were familiarized with the procedures involved in the evaluations. Additionally, match internal and external load variables were monitored for each participant during 4 testing sessions. Each participant performed 4 recreational 6v6 (66.6 m^2^ per player) TH matches, 2 with only same-gender players (i.e., same-gender game format) and 2 with participants of both genders (i.e., mixed-gender game format: 3 men and 3 women), resulting in a total of 20 matches to be analysed. The same- and mixed-gender matches were performed in an indoor TH court (40x20 m). There were 48 hours between each testing session and the participants were asked to refrain from intense physical activity in the 48h before the testing sessions. All testing sessions were performed in the morning. To ensure the maintenance of proper hydration throughout the testing sessions, all participants were instructed to be hydrated and to drink water *ad libitum*.

Each testing session started with a standardized 15-min warm-up (consisting of running, coordination, strength, flexibility, and balance exercises), followed by 3x15-min periods of recreational TH playing, interspersed by 2-min breaks. Some adaptations to the TH rules were made, such as, no body contact was allowed, and softer and lighter TH balls (47 cm circumference, GOALCHA, Fredericia, Denmark) than the official ones were used during the matches. There were some exceptions to the official TH rules, namely, no exclusions, no substitutions, no dribbling. Furthermore, the participants rotated positions every 2 min in a random order, including the goalkeeper, and the ball was immediately put back in play by the goalkeeper after a goal. All training sessions were instructed by a professional TH coach and physical education teacher and monitored by the research team. All the data collection and analysis were performed by the research team that comprised an experienced group of Sport Science, Physical Exercise and Health and Physical Education Teaching Master and PhD graduates.

Participants’ internal load was evaluated as exercise HR, BL concentrations and differential rating of perceived exertion (RPE). Fun levels were also registered at the end of all testing sessions. External load was evaluated as frequency (n), percentage of total match duration (%) and, absolute (m) and relative (%) match distance covered in selected locomotor arbitrary categories [3], considering this study participants’ individual speed thresholds, in order to account for inter-individual variability in external load, as well, as total distance covered.

### Experimental procedures

Body (kg) and fat mass (%) were measured in a bioimpedance digital scale (Tanita Inner Scan BC 532, Tokyo, Japan) and stature (cm) was determined using a portable stadiometer (Seca 213, Hamburg, Germany), according to standardized protocols [11]. BMI was calculated as kg/m^2^. Aerobic performance was evaluated by the YYIE1. The YYIE1 test was performed in the same indoor TH wooden floor court as the matches, according to a protocol already described [11].

The cardiovascular load was monitored in the four testing sessions for each participant. For this purpose, the participants wore a HR monitor (Firstbeat Technologies Ltd., version 4.5.0.2, Jyväskylä, Finland) and their differential RPE (i.e., respiratory, muscular, and global) [21] and fun levels [22] were recorded immediately after the end of each testing session [23]. The participants were familiarised with the considered psychometric scales in training sessions performed before this study. Individual HR_max_ was determined as the highest value reached either during a VO_2max_ test, the YYIE1 test or the matches, according to a multiple testing approach [24]. Moreover, capillary blood samples (30 μl) were drawn from the right earlobe at rest and during the last 5 min of the first and third match periods by a portable electroenzymatic lactate device analyser (Lactate Pro 2 LT-1730, Arkray, Amsterdam, The Netherlands), for measurements of mean and peak BL concentrations. Each of the 41 participants were evaluated 4 times during the study (2 during same- and 2 during mixed-gender matches).

External load was evaluated as match activities variables using time-motion analysis performed by video recordings (SONY-DCR-SX65E, digital video camera recorder) and accelerometer variables using Catapult MinimaxX S4 units (MinimaxX S4; Catapult Sports, Canberra, Australia). Frequency of the selected high-intensity match actions, i.e., jumps, throws, stops, changes of direction and one-on-one situations, and total number of actions were registered via video-analysis of the matches during 20 sessions (4 sessions per participant). Players’ displacements were divided into eight locomotor categories: 1) standing still, 2) walking, 3) jogging, 4) fast running, 5) sprinting, 6) sideways medium-intensity, 7) sideways high-intensity, and 8) backwards movement [3]. High-intensity movements were the result of the sum of fast running, sprinting and sideways high-intensity categories. Individual speed thresholds were considered to determine the individual nature of the exercise intensity in each locomotor category [25] and were registered according to the protocol already described [11]. A test-retest analysis was performed for the selected time-motion variables in 8 matches (4 same-gender and 4 mixed-gender; randomly selected) and the analysis was initiated when intraclass correlation coefficient was >0.80. Table 2 shows the average speed thresholds calculated for each gender in the studied population. Accelerometer data was collected using Catapult MinimaxX S4 units in indoor mode with global positioning system technology in inactive mode. Data was downloaded and processed using Catapult Sprint Version 5.1.1 (Catapult Innovations, Canberra, Australia). The units were located in a specific vest on players’ upper back. The validity and reliability of the accelerometers have been described elsewhere [26]. Player load (an estimate of the physical demand combining the instantaneous rate of change in acceleration in 3 planes [27]) variables were evaluated at a 100 Hz sampling rate. In this study, time spent in each player load zone, i.e., 0–0.1, ˃0.1–0.3, ˃0.3–0.6, ˃0.6–1.0, ˃1.0–1.5, ˃1.5–2.0, ˃2.0 [27] was presented as percentage of total match time, whereas total accumulated player load was presented as arbitrary units [26]. Frequency of accelerations and decelerations, categorized as low (1.50 to 2.14 m.s^-1^), medium (2.14 to 2.78 m.s^-1^) and high-intensity (˃2.78 m.s^-1^), according to manufacture settings (Catapult Sprint Version 5.1.1 software manual, Catapult Innovations, Canberra, Australia), was determined.

**Table 2:** Player’s locomotor speed categories according to gender (average values for this population).

Locomotor category	Men	Women
Standing	0 km h^-1^	0 km h^-1^
Walking	6 km h^-1^	6 km h^-1^
Jogging	9 km h^-1^	8 km h^-1^
Fast running	12 km h^-1^	11 km h^-1^
Sprinting	17 km h^-1^	13 km h^-1^
Sideways medium-intensity movements	8 km h^-1^	6 km h^-1^
Sideways high-intensity movements	10 km h^-1^	7 km h^-1^
Backwards movements	8 km h^-1^	5 km h^-1^

### Statistical analyses

Results are presented as means ± standard deviations (SD) and 95% of confidence interval (CI). Students’ unpaired t-test was used to assess differences between genders in chronological age, stature, body composition, BMI, aerobic performance and time experience with recreational TH. To examine differences between the genders during the same- and mixed-gender matches a two-way analysis of variance (ANOVA) for repeated measures with Bonferroni post hoc multiple comparison tests was used. Power calculations were performed to detect an effect size of 0.18 in repeated measures ANOVA (within factors only). With 2 measurements, alpha of 5%, and power of 80%, 39 participants were needed. Effect size was calculated using Cohen *d* and interpreted as trivial (˂0.2), small (0.2–0.5), medium (0.5–0.8) and large (>0.8) [28]. Relative reliability of key HR variables was reported as Intraclass Correlation Coefficients (ICC_3,1_). Magnitude of the ICC values was rated as excellent, good, and poor for 0.75–1.00, 0.41–0.74, and 0.00–0.40 scores, respectively [29,30]. Statistical Package for the Social Sciences (SPSS Inc., version 23.0) was used for the analyses. The data were tested for normality using the Shapiro-Wilk test. Statistical significance was set at *p≤*0.05.

## Results

### Internal load and perceived experience

**Fig 1:**
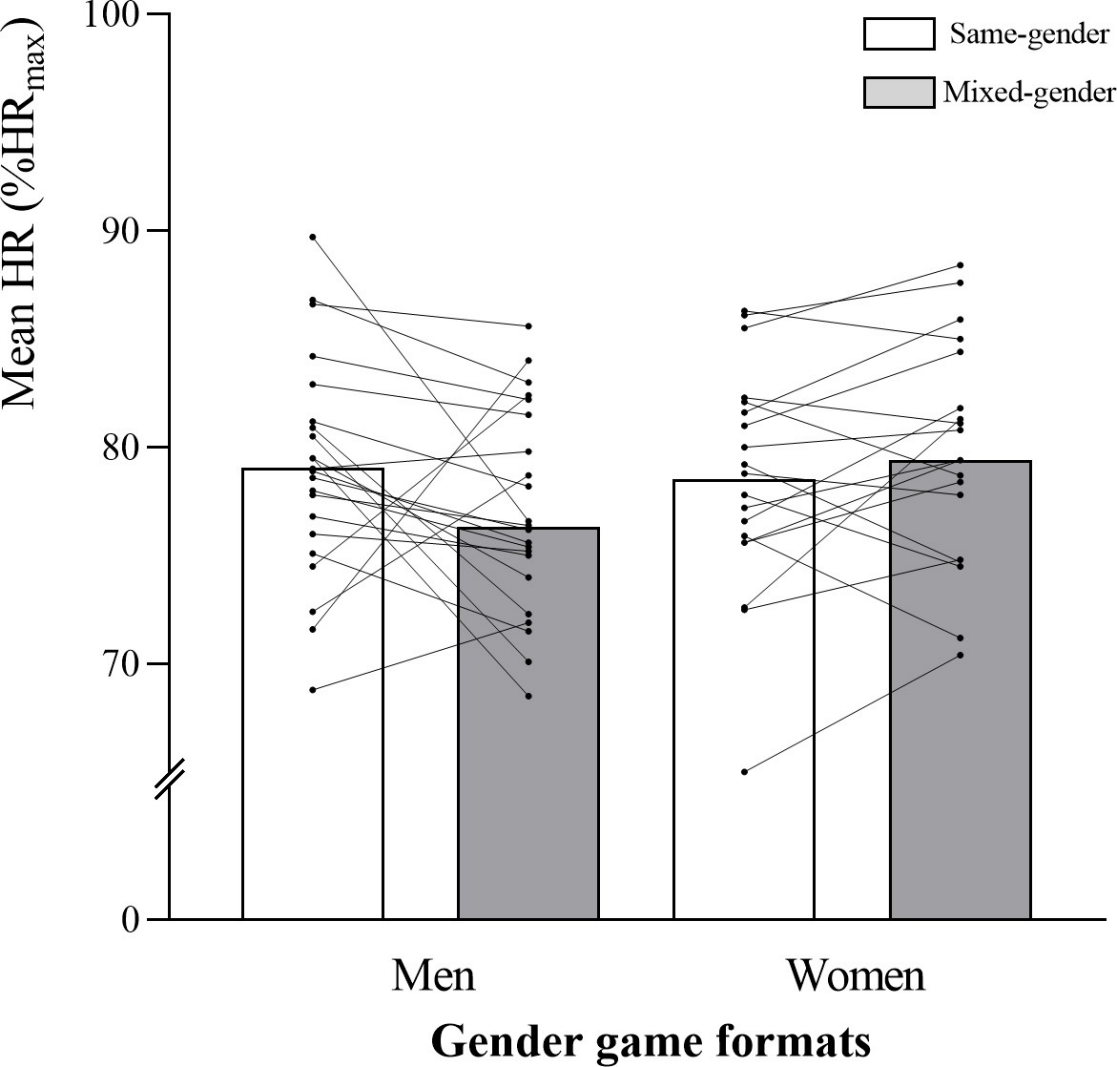
Mean heart rate (mean HR) for men and women in each gender game format (same- and mixed-gender). Data are presented as means±SD.

**Fig 2:**
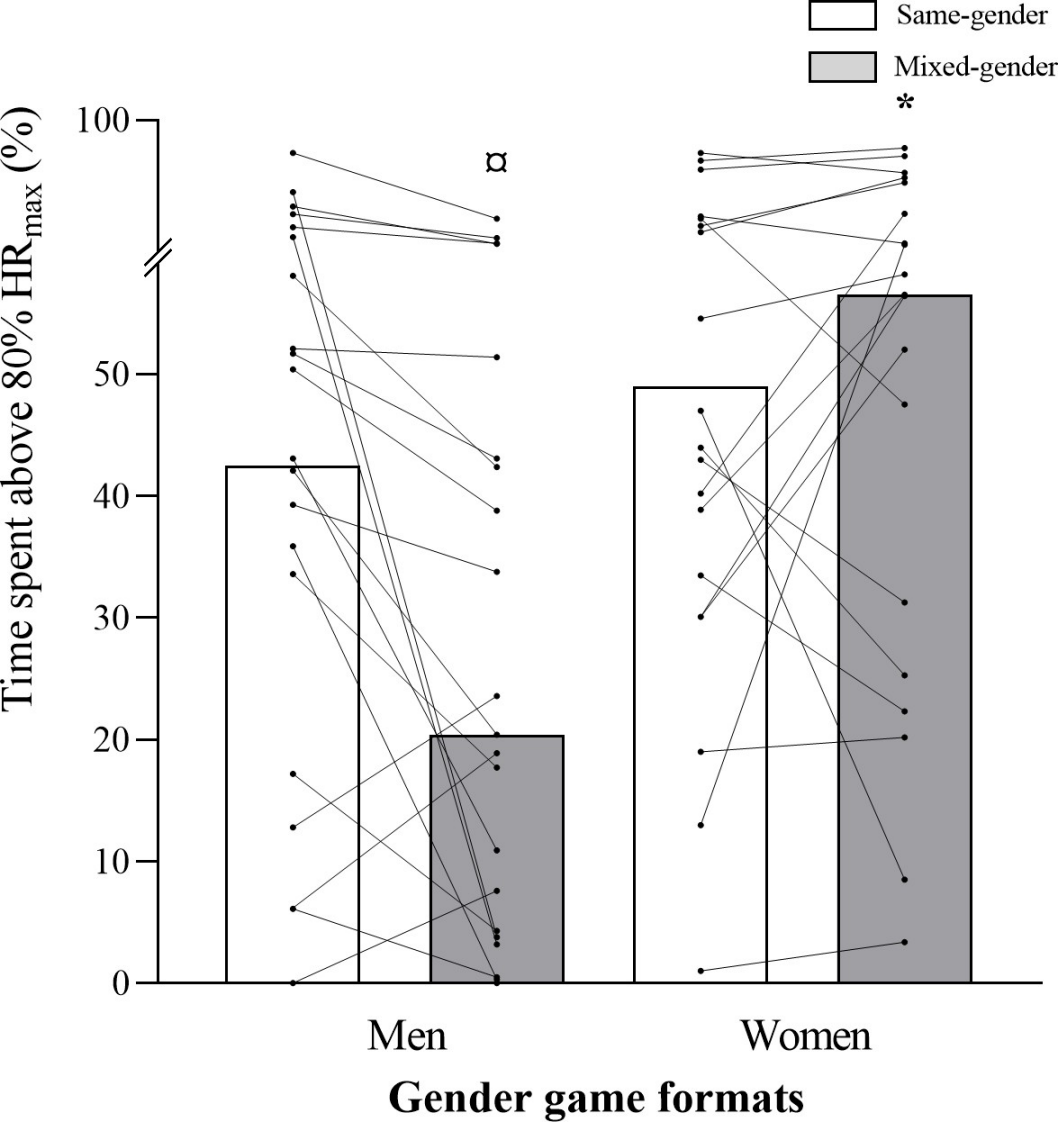
Percentage of total match time spent with heart rates above 80% of individual maximal HR (%HR_max_) for men and women in each gender game format (same- and mixed-gender). Data are presented as means±SD. ^*^Significantly different from Men Mixed-Gender; ^¤^Significantly different from Men Same-Gender.

**Fig 3:**
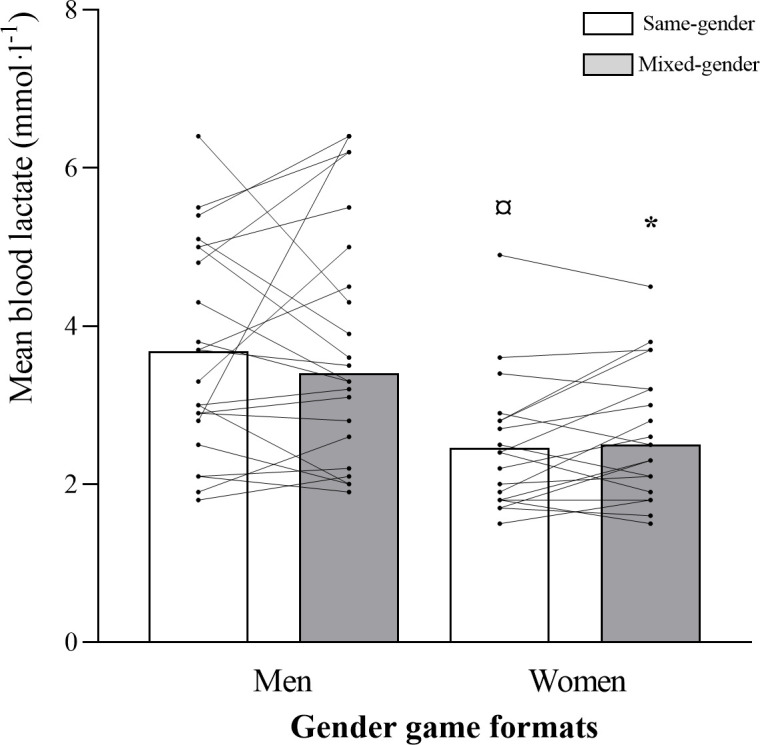
Mean blood lactate (mmol·l^-1^) values for men and women in each gender game format (same- and mixed-gender). Data are presented as means±SD. ^*^Significantly different from Men Mixed-Gender; ^¤^Significantly different from Men Same-Gender.

**Fig 4:**
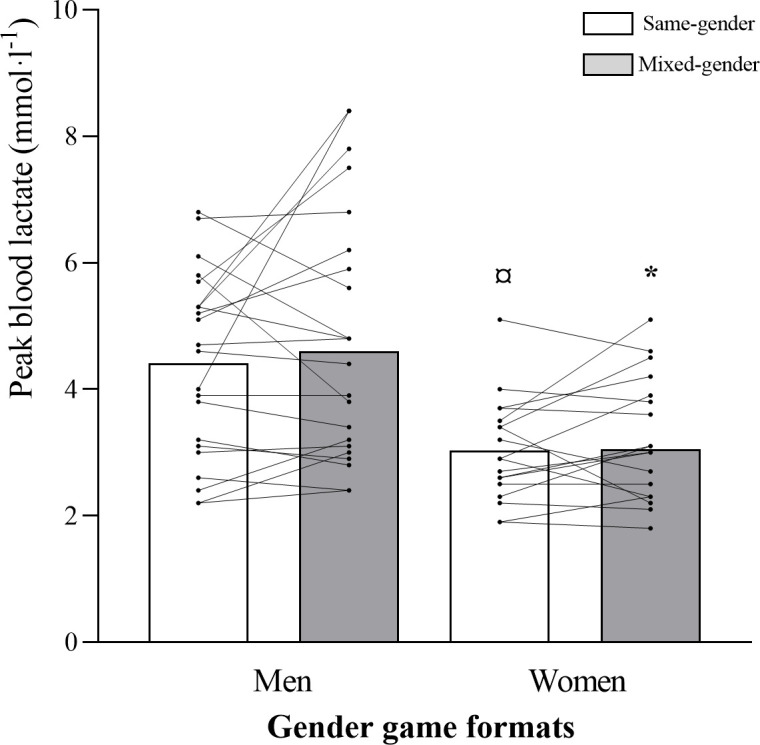
Peak blood lactate (mmol·l^-1^) values for men and women in each gender game format (same- and mixed-gender). Data are presented as means±SD. ^*^Significantly different from Men Mixed-Gender; ^¤^Significantly different from Men Same-Gender.

A game format x gender interaction was observed for relative mean HR (*p =* 0.043; Table 3 and Fig 1), for the percentage of time spent ˃80%HR_max_ (*p =* 0.051) and ˃90%HR_max_ (*p =* 0.011), and for respiratory RPE (*p =* 0.032). Percentages of time spent ˃80%HR_max_ (*p =* 0.008; 95% CI: -39.96, -6.25; *d =* 0.360) and ˃90%HR_max_ (*p =* 0.017; 95% CI: -19.41, -2.06; *d =* 0.451) were higher for women than for men, while playing mixed-gender matches. Men’s percentage of time spent ˃80%HR_max_ was higher during same- vs. mixed-gender matches (*p =* 0.034; 95% CI: -0.53–23.29; *d =* 0.141; Fig 2), while women’s percentage of time spent ˃90%HR_max_ was lower during same- vs. mixed-gender matches (*p =* 0.036; 95% CI: -12.86–0.44; *d =* 0.459). Women’s BL values were lower than men’s during same- (mean BL: *p =* 0.002; 95% CI: 0.49–1.92; *d =* 0.980; peak BL: *p =* 0.011; 95% CI: 0.28–2.02; *d =* 0.999; first period BL: *p≤*0.001; 95% CI: 0.61–2.08; *d =* 1.179; third period BL: *p =* 0.008; 95% CI: 0.29–1.85; *d =* 0.980) and mixed-gender matches (mean BL: *p =* 0.005; 95% CI: 0.37–1.97; *d =* 0.972; peak BL: *p =* 0.015; 95% CI: 0.29–2.48; *d =* 0.887; first period BL: *p =* 0.009; 95% CI: 0.34–2.19; *d =* 1.094; third period BL: *p =* 0.010; 95% CI: 0.27–1.88; *d =* 0.743) (Figs 3 and 4). No significant differences were observed for muscular and global RPE and fun levels between the gender game formats. In men, the ICC across the same- and mixed-gender game formats for mean HR, time ˃80% HR_max_, and time ˃90% HR_max_ were 0.61 (0.18–0.84, good), 0.51 (0.12–0.76, good), and 0.35 (0.18–0.84, poor), respectively. For mean HR, time ˃80% HR_max_, and time ˃90% HR_max_, the ICC values for the different gender game formats in the female participants were 0.77 (0.50–0.91, excellent), 0.67 (0.31–0.86, good), and 0.72 (0.40–0.88, good), respectively.

**Table 3:** Men and women’s internal load during 6v6 same- and mixed-gender recreational team handball game formats (data are presented as mean ± SD).

	Men		Women		Game format	Gender	Interaction
	Same- gender	Mixed-gender	Same-gender	Mixed-gender			
	(n = 22)		(n = 19)		*p*	*p*	*p*
**Cardiovascular demands**							
Mean HR (%HR_max_)	78±5	76± 5	76±8	79±6	0.617	0.427	0.043
Peak HR (%HR_max_)	86±6	85±6	88±6	88±6	0.498	0.340	0.150
Time ˃80% HR_max_ (%)	36±28	32±28^¤^	44± 32	47±33^*^	0.332	0.043	0.051
Time ˃90% HR_max_ (%)	7±16	3±5	5±5	9±18^*^^#^	0.614	0.136	0.011
Time ≤60% HR_max_ (%)	1±2	2±3	9±13	1±2	0.923	0.565	0.104
Time 61–70% HR_max_ (%)	17±15	19±12	21±17	14±15	0.728	0.101	0.299
Time 71–80% HR_max_ (%)	47±21	48±18	27±7	38±18^*^	0.261	0.083	0.231
Time 81–90% HR_max_ (%)	29±17	29±25	39±28	38±24	0.201	0.059	0.503
**BL concentrations**							
First period mean BL (mmol·l^-1^)	4.0±1.4	4.1±1.8	2.5±0.5^¤^	2.5±0.7^*^	0.344	0.001	0.820
Third period mean BL (mmol·l^-1^)	3.0±1.5	3.1±1.6	1.7±0.5^¤^	1.9±0.8^*^	0.426	0.004	0.989
Match mean BL (mmol·l^-1^)	3.5±1.7	3.6±1.6	2.1±0.5^¤^	2.2±0.7^*^	0.284	0.001	0.895
Match peak BL (mmol·l^-1^)	4.2±1.5	4.5±2.0	2.8±0.7^¤^	2.9±0.9^*^	0.103	0.008	0.529
**RPE**							
Respiratory RPE (AU, 0–10)	6.8±1.8	5.9±2.2	5.9±1.8	6.1±2.9	0.918	0.948	0.032
Muscular RPE (AU, 0–10)	6.8±1.7	6.0±2.0	5.6±2.1	5.3±2.5	0.709	0.543	0.206
Global RPE (AU, 0–10)	6.6±1.8	5.6±2.5	5.9±1.7	5.9±2.6	0.659	0.991	0.069
**Fun** (AU, 0–10)	9.3±0.9	9.1±1.1	9.3±1.0	9.1±1.5	0.811	0.849	0.335

AU—Arbitrary units; BL–Blood lactate; HR—Heart rate; HR_max_−Maximal heart rate; RPE—Rating of perceived exertion.

^*^Significantly different from Men Mixed-Gender

^#^Significantly different from Women Same-Gender

^¤^Significantly different from Men Same-Gender.

### External load

#### Locomotor activity profile

**Fig 5:**
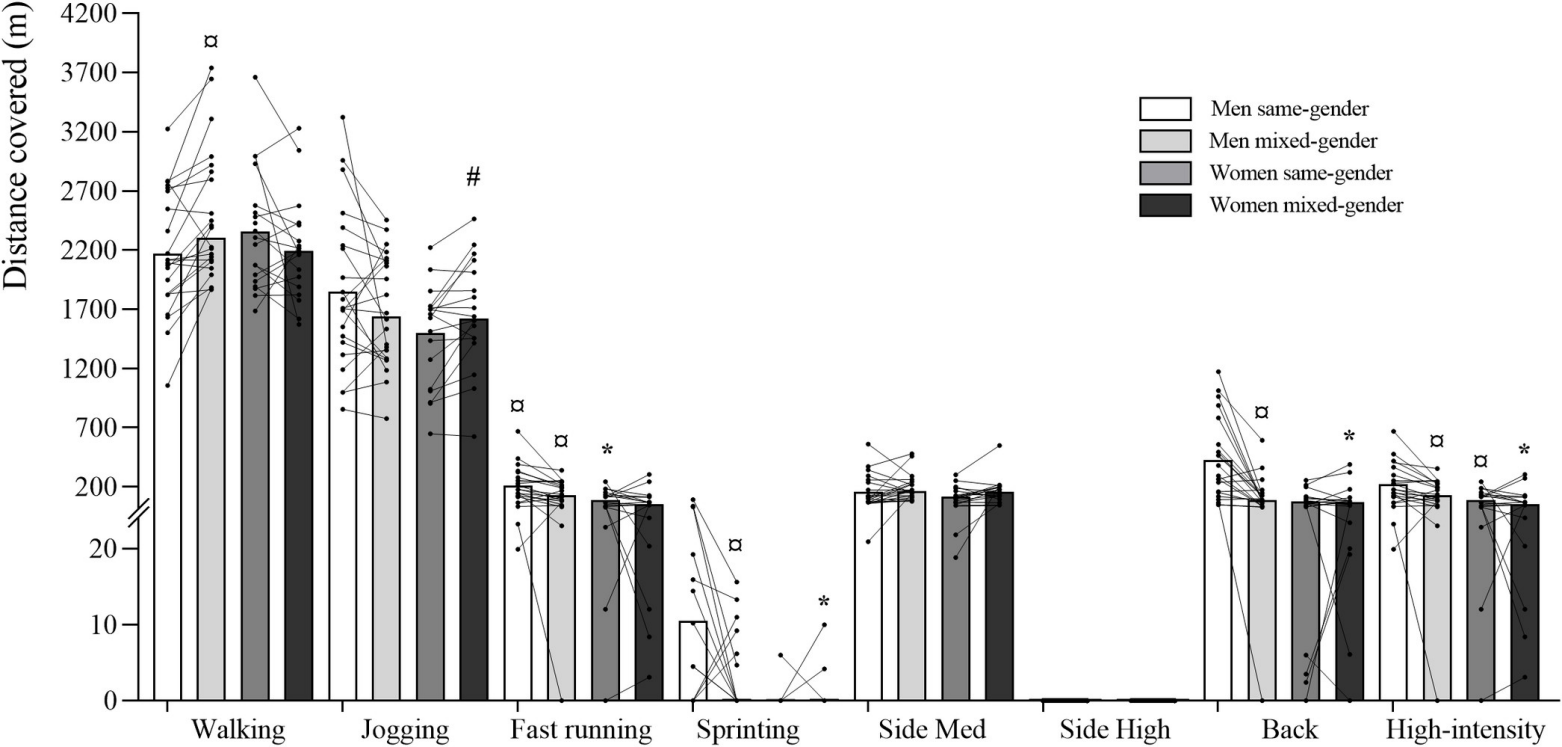
Distance covered (m) for the locomotor activity categories in each gender game format (same- and mixed-gender). Data are presented as means±SD. Back–backwards movements; High-intensity–sum of fast running, sprinting and sideways high-intensity movements; Side High–sideways high-intensity movements; Side Med–sideways medium-intensity movements. ^*^Significantly different from Men Mixed-Gender; ^#^Significantly different from Women Same-Gender; ^¤^Significantly different from Men Same-Gender.

Participants’ locomotor activity profile is presented in Table 4 and Fig 5. A game format x gender interaction was found for frequency of standing, walking, sprinting, and backwards movements (*p≤*0.048), for the percentage of total match duration spent standing, walking, jogging and in backwards movements (*p≤*0.020), for the absolute and percentage of total match distance covered jogging and in backwards movements (*p≤*0.037) and for the percentage of total match distance covered walking (*p =* 0.010). A game format effect was observed for frequency of standing, fast running, sideways medium-intensity, backwards and high-intensity movements (*p≤*0.050; Table 4), for percentage of total match duration in sideways medium-intensity and backwards movements (*p≤*0.031) and for absolute and percentage of total match distance covered fast running, and in sideways medium-intensity, backwards and high-intensity movements (*p≤*0.022).

**Table 4:** Men and women’s locomotor activity profile during 6v6 same- and mixed-gender recreational team handball game formats (data are presented as mean ± SD).

Locomotor categories	Men		Women				
	Same-Gender	Mixed-Gender	Same-Gender	Mixed-Gender	Game format	Gender	Interaction
	(n = 22)		(n = 19)		*p*	*p*	*p*
**Freq (n)**							
Standing	12±8	21±5^¤^	17±6^¤^	16±7^*^	0.038	0.913	˂0.001
Walking	94±17	101±10	93±8	92±35	0.645	0.459	0.048
Jogging	74±18	74±14	73±13	75±30	0.965	0.747	0.526
Fast running	13±8	10±6	6±4^¤^	5±2^*^	0.050	0.002	0.500
Sprinting	0.8±1.4	0.1±0.3^¤^	0.0±0.0	0.0±0.0^*^	0.180	0.025	0.043
Side Med	15±9	18±8^¤^	12±6	15±7	0.009	0.548	0.276
Side High	0.0±0.0	0.0±0.0	0.0±0.0	0.0±0.0			
Back	30±17	10±6^¤^	10±8	9±6^*^	˂0.001	0.008	˂0.001
High-intensity	14±9	10±6^¤^	6±4^¤^	5±2^*^	0.050	0.001	0.355
Total	240±42	234±30	211±23	212±75	0.744	0.084	0.371
**Total match duration (%)**							
Standing	9±7	14±5^¤^	17±5	15±2^*^	0.158	˂0.001	0.020
Walking	50±10	55±6^¤^	54±6^¤^	50±4^#^	0.706	0.366	0.001
Jogging	28±8	24±6^¤^	25±6	28±3^#^	0.915	0.904	0.002
Fast running	2.2±1.4	1.6±1.1	1.0±0.7	1.5±2.6^*^	0.739	0.037	0.092
Sprinting	0.1±0.2	0.0±0.0^¤^	0.0±0.0	0.0±0.0^*^	0.209	0.048	0.070
Side Med	3.1±2.4	3.4±2.0	2.2±1.3	3.3±1.5	0.031	0.703	0.586
Side High	0.0±0.0	0.0±0.0	0.0±0.0	0.0±0.0			
Back	7.7±.5	2.0±1.5^¤^	2.0±1.8	1.9±1.6^*^	˂0.001	0.006	˂0.001
High-intensity	2.3±1.5	1.6±1.1	1.0±0.7	1.5±2.6^*^	0.675	0.032	0.076
**Total match distance (m)**							
Walking	2203±443	2534±501^¤^	2433±579	2456±1047	0.107	0.875	0.080
Jogging	1804±697	1614±494	1319±375	1724±762^#^	0.499	0.374	0.010
Fast running	231±165	147±102^¤^	93±74^¤^	56±30^*^	0.016	0.003	0.088
Sprinting	12.8±24.1	2.0±4.8^¤^	0.0±0.0	0.0±0.0^*^	0.169	0.059	0.057
Side Med	183±143	203±116	89±48	152±66	0.020	0.251	0.669
Side High	0.0±0.0	0.0±0.0	0.0±0.0	0.0±0.0			
Back	442±339	101±82^¤^	64±60	70±53^*^	˂0.001	˂0.001	˂0.001
High-intensity	244±175	149±105^¤^	93±74^¤^	56±30^*^	0.014	0.003	0.061
Total	4875±713	4601±629	3998±671	4457±1729^*^	0.674	0.063	0.084
**Total match distance (%)**							
Walking	46±13	55±9^¤^	61±8	55±8^*^^#^	0.438	0.478	0.010
Jogging	36±10	35±9	33±7	39±7^#^	0.421	0.835	0.037
Fast running	5±3	3±2^¤^	2±2^¤^	1±1^*^	0.022	0.005	0.232
Sprinting	0.3±0.5	0.0±0.1^¤^	0.0±0.0	0.0±0.0	0.162	0.062	0.065
Side Med	4±3	4±3^¤^	2±1	4±2	0.009	0.491	0.973
Side High	0.0±0.0	0.0±0.0	0.0±0.0	0.0±0.0			
Back	9±6	2±2^¤^	2±2	2±1^*^	˂0.001	0.001	˂0.001
High-intensity	5±3	3±2^¤^	2±2^¤^	1±1^*^	0.018	0.004	0.163

Back–backwards movements; Freq–Frequency; High-intensity–sum of fast running, sprinting and sideways high-intensity movements; Side High–sideways high-intensity movements; Side Med–sideways medium-intensity movements.

^*^Significantly different from Men Mixed-Gender

^#^Significantly different from Women Same-Gender

^¤^Significantly different from Men Same-Gender.

Men´s frequency of high-demanding movements was higher during same- vs. mixed-gender matches (sprinting: *p =* 0.015; 95% CI: -1.02–0.02; *d =* 0.707; high-intensity: *p =* 0.036; 95% CI: -5.88–0.43; *d =* 0.558). During same-gender game formats, men’s frequency of fast running (*p =* 0.005; 95% CI: 1.89–9.66; *d =* 0.960) and high-intensity movements was also higher than during mixed-gender matches (*p =* 0.006; 95% CI: 2.23–10.72; *d =* 1.011). During mixed-gender matches the frequency of fast running (*p =* 0.005; 95% CI: 1.50–7.80; *d =* 1.038), sprinting (*p =* 0.019; 95% CI: -0.18–0.37; *d =* 0.599) and in high-intensity movements (*p =* 0.004; 95% CI: 1.45–8.05; *d =* 1.013) was higher for men than in women.

Men’s percentage of total match duration spent standing (*p =* 0.007; 95% CI: 0.78–7.06; *d =* 0.663) and walking (*p =* 0.008; 95% CI: 0.80–7.84; *d =* 0.740) was higher when playing mixed- vs. same-gender matches, however, in jogging (*p =* 0.018; 95% CI: -6.31–0.09; *d =* 0.576), sprinting (*p =* 0.026; 95% CI: -0.12–0.01; *d =* 0.958) and backwards movements (*p˂*0.001; 95% CI: -7.49, -2.83; *d =* 1.455) was lower during mixed- vs. same-gender matches. During mixed-gender game formats, men’s percentage of total match duration spent fast running (*p =* 0.002; 95% CI: -0.65–1.27; *d =* 0.632), sprinting (*p =* 0.042; 95% CI: -0.02–0.04; *d =* 0.00), in backwards (*p˂*0.001; 95% CI: -1.67–1.35; *d =* 0.005) and high-intensity movements (*p =* 0.002; 95% CI: -0.64–1.29; *d =* 0.004) was higher than for women. For women, the percentage of total match duration spent walking (*p =* 0.044; 95% CI: -6.50, -0.41; *d =* 0.769) was higher, and jogging was lower (*p =* 0.038; 95% CI: 1.05–4.77; *d =* 0.928) during same- vs. mixed-gender matches.

During mixed-gender matches, men’s absolute match distance covered fast running (*p =* 0.004; 95% CI: 11.71–121.83; *d =* 1.022), sprinting (*p =* 0.046; 95% CI: -2.46–4.63; *d =* 0.627), and in backwards (*p˂*0.001; 95% CI: -39.95–111.51; *d =* 0.438) and high-intensity movements (*p =* 0.003; 95% CI: 10.98–124.73; *d =* 1.023) as well as total distance (*p =* 0.003; 95% CI: -425.52–826.37; *d =* 0.098) were higher than women’s. Men’s absolute and percentage of total match distance covered with high-intensity movements, namely, fast running (absolute: *p =* 0.003; 95% CI: -111.60, -18.06; *d =* 0.888; percentage: *p =* 0.012; 95% CI: -2.11, -0.15; *d =* 0.970) and sprinting (absolute: *p =* 0.018; 95% CI: -16.36–0.74; *d =* 0.778; percentage: *p =* 0.019; 95% CI: -0.33–0.02; *d =* 1.125) was higher when playing same- vs. mixed-gender matches. For women, percentage of total match distance covered walking was higher (*p =* 0.037; 95% CI: -7.10, -0.99; *d =* 1.106), and jogging was lower (*p =* 0.049; 95% CI: 0.13–6.26; *d =* 1.016) during same- vs. mixed-gender matches. During mixed-gender matches, the percentage of total match distance covered walking (*p =* 0.003; 95% CI: -5.57–6.32; *d =* 0.019), fast running (*p =* 0.008; 95% CI: 0.23–2.46; *d =* 1.096), and in backwards (*p˂*0.001; 95% CI: -0.89–2.13; *d =* 0.006), and high-intensity movements (*p =* 0.006; 95% CI: 0.23–2.52; *d =* 1.102) was higher for men than women. During same-gender matches, the absolute and percentage of total match distance covered fast running (absolute: *p =* 0.019; 95% CI: 41.95–198.25; *d =* 1.063; percentage: (*p =* 0.019; 95% CI: 0.59–3.62; *d =* 1.067) and in high-intensity movements (absolute: *p =* 0.021; 95% CI: 48.11–212.51; *d =* 1.141; percentage: (*p =* 0.021; 95% CI: 0.71–3.91; *d =* 1.086) was higher for men than for women.

#### High-intensity game actions

A game format x gender interaction was observed for the frequency of throws (*p =* 0.045; Table 5). During mixed-gender matches, frequency of jumps (*p =* 0.009; 95% CI: 1.07–5.05; *d =* 1.501; Table 5), stops (*p =* 0.008; 95% CI: 0.06–4.45; *d =* 0.598), changes of direction (*p =* 0.004; 95% CI: -0.07–3.16; *d =* 1.020) and total high-intensity game actions (*p =* 0.014; 95% CI: 4.44–18.16; *d =* 0.712) was higher for men than for women. During same-gender matches, men showed higher frequency of jumps (*p =* 0.004; 95% CI: 0.77–4.96; *d =* 0.622), throws (*p =* 0.005; 95% CI: -2.21–2.84; *d =* 0.537), stops (*p =* 0.044; 95% CI: 0.89–5.51; *d =* 2.104), one-on-one situations (*p =* 0.028; 95% CI: -0.53–2.59; *d =* 0.546) and total high-intensity game actions (*p =* 0.002; 95% CI: 2.30–19.00; *d =* 0.727) than women.

**Table 5:** Men and women’s high-intensity game actions during 6v6 same- and mixed-gender recreational team handball game formats (data are presented as means ± SD).

Game actions/gender game format	Men		Women				
	Same-Gender	Mixed-Gender	Same-Gender	Mixed-Gender	Game format	Gender	Interaction
	(n = 22)		(n = 19)		*p*	*p*	*p*
Jumps (n)	9±0	7±2	6±7^¤^	2±3^*^	0.402	0.002	0.829
Throws (n)	11±1	9±1	8±7^¤^	6±3	0.947	0.121	0.045
Stops (n)	16±2	15±8	8±5^¤^	11±4^*^	0.161	0.007	0.414
Changes of direction (n)	12±4	12±5	8±4	9±2^*^	0.332	0.007	0.063
One-on-one situations (n)	11±0	12±5	8±8^¤^	7±4	0.182	0.029	0.399
Total high-intensity actions (n)	43±15	44±11	32±11^¤^	33±11^*^	0.363	0.003	0.841

^*^Significantly different from Men Mixed-Gender;

^¤^Significantly different from Men Same-Gender.

#### Player load and accelerometer data

A game format x gender interaction was observed for time spent in ˃0.3–0.6% player load zone (*p =* 0.042; Table 6). During same- and mixed-gender matches, time spent in 0.0–0.1% player load zone was lower for men than for women (same-gender: *p =* 0.004; 95% CI: -13.66, -5.05; *d =* 2.048; mixed-gender: *p˂*0.001; 95% CI: -9.28, -1.91; *d =* 1.232, respectively; Table 6). However, time spent in ˃0.1–0.3 (same-gender: *p =* 0.022; 95% CI: 0.43–8.22; *d =* 0.898; mixed-gender: *p =* 0.031; 95% CI: 0.74–8.99; *d =* 1.323), ˃1.5–2.0 (same-gender: *p˂*0.001; 95% CI: 1.97–5.61; *d =* 8.276; mixed-gender: *p˂*0.001; 95% CI: 1.39–5.34; *d =* 5.183) and ˃2.0% (same-gender: *p =* 0.002; 95% CI: 0.62–3.05; *d =* 5.484; mixed: *p =* 0.004; 95% CI: 0.95–3.47; *d =* 6.458) player load zones and total accumulated player load (same-gender: *p =* 0.034; 95% CI: 9.77–78.75; *d =* 1.494; mixed-gender: *p =* 0.013; 95% CI: 3.13–73.34; *d =* 2.589) was higher for men than for women during same- and mixed-gender matches. Men’s low-intensity (*p =* 0.012; 95% CI: -4.70–22.49; *d =* 0.981) and total accelerations (*p =* 0.040; 95% CI: -0.97–37.80; *d =* 1.735) were higher than women’s during mixed-gender matches. During same-gender matches, medium (*p =* 0.014; 95% CI: -0.87–5.87; *d =* 2.196) and high-intensity (*p =* 0.005; 95% CI: -1.29–6.71; *d =* 3.541) accelerations were higher for men than for women. No significant differences were found for decelerations.

**Table 6:** Men and women’s player load and accelerometer data during 6v6 same- and mixed-gender recreational team handball game formats (data are presented as mean ± SD).

Accelerometer variables/gender game formats	Men		Women					
	Same-Gender	Mixed-Gender	Same-Gender	Mixed-Gender		Game format	Gender	Interaction
	(n = 22)		(n = 19)			*p*	*p*	*p*
**Player load zones**								
Time 0.0–0.1 (%)	12±6	17±3	25±4^¤^		22±2^*^	0.511	˂0.001	0.082
Time ˃0.1–0.3 (%)	37±4	40±0	34±1^¤^		36±5^*^	0.577	0.018	0.694
Time ˃0.3–0.6 (%)	21±1	19±2	20±4		23±6^#^	0.129	0.773	0.042
Time ˃0.6–1.0 (%)	9±2	8±2	8±4		7±3	0.206	0.414	0.448
Time ˃1.0–1.5 (%)	10±2	11±1	11±5^¤^		10±6	0.611	0.131	0.169
Time ˃1.5–2.0 (%)	8±0	5±0	2±0^¤^		2±1^*^	0.275	˂0.001	0.554
Time ˃2.0 (%)	2±1	1±0	0±0^¤^		0±0^*^	0.404	0.001	0.478
Total accumulated (AU)	353±22	317±4	278±51^¤^		263±29^*^	0.533	0.008	0.740
**Accelerations**								
Low-intensity (n)	24±6	14±7	10±6		10±0^*^	0.829	0.042	0.721
Medium-intensity (n)	14±5	12±3	6±1^¤^		7±0	0.115	0.016	0.708
High-intensity (n)	18±6	17±8	6±0^¤^		5±1	0.673	0.006	0.182
Total (n)	55±17	43±18	21±6		22±1^*^	0.753	0.011	0.531
**Decelerations**								
Low-intensity (n)	13±0	14±5	11±9		7±8	0.982	0.734	0.962
Medium-intensity (n)	6±0	7±5	6±6		5±4	0.431	0.479	0.737
High-intensity (n)	6±5	8±5	21±28		2±1	0.094	0.627	0.841
Total (n)	24±6	29±14	38±43		13±13	0.275	0.598	0.951

AU–arbitrary units.

^*^Significantly different from Men Mixed-Gender

^#^Significantly different from Women Same-Gender

^¤^Significantly different from Men Same-Gender.

## Discussion

This is the first study analysing the internal and external load of same- vs. mixed-gender recreational TH game formats for middle-aged and elderly men and women. Game format-by-gender interactions were found for relative mean HR, time spent >80%HR_max_ and ˃90%HR_max_ and respiratory RPE, and also for several of the external load variables considered. The main findings were that during mixed-gender matches, time spent ˃80%HR_max_ and ˃90%HR_max_ was higher for women than men, while mean and peak BL values were lower. Furthermore, in same-gender matches, men’s time spent with HR ˃80%HR_max_, as well as several activity profile variables, such as high-intensity locomotor movements were higher than in mixed-gender matches.

Studies using recreational TH as an exercise intervention have shown that this exercise mode is effective in inducing several health improvements in different age groups and in both genders [4–9]. It has also been shown that the time spent in high HRs zones (i.e., HR >90%HR_max_) is positively associated (r = 0.61) with VO_2max_ improvement [7]. Moreover, studies using recreational football have also suggested that time spent with HR ˃90% HR_max_ (˜20% of total match time) was the possible cause of the reported improvements in VO_2max_ [31]. In the present study, the participants spent less time with HR ˃90%HR_max_ (3–9% of total match time) then the studies reported above. Nevertheless, improvements in VO_2max_ were observed in postmenopausal women by spending around 11% of total match time in HR ˃90%HR_max_ [4], meaning that for this age population, spending less than 20% of total match time in HR ˃90%HR_max_ is still able to induce improvements in cardiorespiratory fitness.

In the present study, during mixed-gender matches, time spent ˃80% and ˃90%HR_max_ was higher for women than for men. This may indicate that during mixed-gender matches, women increase their physical demands in order to keep up with the pace imposed by men. In fact, the ability to perform high-intensity intermittent activity was higher in the male vs. female participants (YYIE1: 754±439 vs. 395±158 m, men and women, respectively). Yo-Yo tests’ performance has been positively associated with the amount of high-intensity running during football matches [32]. Men’s performance superiority was observed in the matches’ external demands. In fact, during same- and mixed-gender matches, the frequency of high-intensity movements and specific high-intensity game actions, the time spent in the highest player load zones, the total accumulated player load and frequency of total accelerations were higher for men than for women, which may be the reason why women showed lower mean and peak BL values. Nevertheless, during mixed-gender matches, women showed higher cardiovascular load than men, while performing less external load (e.g., less time spent and lower distance covered in high-intensity movements and lower total distance covered), which can be attributed to their lower aerobic performance level compared to men [32]. During same-gender matches, men’s time spent ˃80%HR_max_ was higher than in mixed-gender matches, which is line with the locomotor activity profile. Men performed higher intensity locomotor movements during same- than during mixed-gender matches, which may explain the higher HRs shown during same-gender matches.

Both during same- and mixed-gender matches, men’s and women’s relative mean and peak HR values were slightly lower than those reported for younger populations [3,5–8]. However, they were in line with those reported for postmenopausal women [4,9], who showed cardiovascular and musculoskeletal health improvements after 16 weeks of recreational TH training with those intensities. Relative reliability is a viable strategy to assess the interindividual consistency from evaluation-to-evaluation, providing information over the underpinning variability within and between the evaluations. In this study, the ICC value of the key HR variables was studied across the gender game formats (i.e., same- vs mixed-games). The results showed poor-to-good match intensity (HR) consistency for the men and good-to-excellent scores for the women. This data suggests individual monitoring of exercise intensity across the gender game formats when men play with their female counterparts. Interestingly, women’s mean and peak BL values were lower than the men’s (3.5–3.6 and 4.2–4.5 mmol·l^-1^, respectively), which could be related with the higher frequency, percentage of total match time and distance covered in high-intensity movements by men compared to women, during both gender game formats. Nevertheless, men’s mean and peak BL values were similar to those reported for middle-aged former TH players (3.6 and 4.2 mmol·l^-1^, respectively) [3]. Based on this study results, playing same- or mixed-gender matches results in internal load values in the range to induce cardiovascular adaptations seen in intervention studies with other age-groups using recreational TH as exercise mode.

During same-gender matches, the lower BL values observed for women when compared to men, could be related with the higher frequency of TH high-demanding game-specific actions such as jumps, throws, stops, one-on-one situations and total high-intensity actions, more time spent in higher player load zones (˃1.5–2.0 and ˃2.0), higher total accumulated player load, and higher frequency of medium and high-intensity accelerations found in men. The BL values were also lower for women in mixed-gender matches, which may have been the result of men also showing higher frequency of TH high-intensity game-specific actions such as jumps, stops, changes of direction and total high-intensity actions, spending more time in higher player load zones (˃1.5–2.0 and ˃2.0), showing higher total accumulated player load and performing more low-intensity and total accelerations than women, indicating that men were more involved in the matches than women and, consequently, had a higher level of participation in the matches. This is important to maintain the participants’ motivation to keep playing [33]. Accordingly, women could have felt less involved and motivated in the game. Nevertheless, this was not the case, since differential RPE were similar for men and women as well as fun levels, which were very high (9.1–9.3 AU on a 0–10 scale) in both gender game formats. Therefore, for this population, both gender game formats could be recommended for both genders, contrary to children, with boys reporting more fun when playing football in same- vs. mixed-gender matches [18].

Our study showed that in recreational TH, men and women spent around 50–55% of the total match time walking and 24–28% jogging. The intensities alternate during a TH game due to its intermittent nature, with the locomotor activity pattern changing from standing and walking, jogging and moderate running, fast running and sprinting, sideways and backwards movements [34,35], requiring a high level of endurance to keep up with the game demands. Nevertheless, TH performance is also highly influenced by cognitive, tactical, and social factors [2]. This may explain the higher amount of time spent jogging (24–28% vs. 16%, respectively) and the less time spent in high-intensity movements (1–2% vs. 15%, respectively), by this study participants, that have a clear lack of knowledge and experience with the sport, when compared to former TH players [3], whose technical-tactical sport background may have allowed them to better manage and control the game than the unexperienced older participants.

From a physiological point of view, in order to achieve the highest intensities, recreational TH interventions should be preferentially organized as same-gender game formats for men and as mixed-gender for women. Nevertheless, men’s and women’s HRs in both same- and mixed-gender TH matches, were in line with studies showing cardiovascular health improvements, which from a practical perspective, means that both gender game formats may be used to induce cardiovascular adaptations for this population. Perhaps, it would be of interest to organize different recreational TH training sessions by changing the gender game formats, allowing men and women to alternate and benefit from different match demands given by the different gender game formats.

### Strengths and limitations

The main strength of this study is that it is the first to analyse the differences in internal and external load variables in both men and women playing same- vs. mixed-gender recreational TH games. This is important since this multicomponent exercise programme is usually implemented in community settings, aiming at men and women, and therefore understanding the physical and physiological demands and the perceived experience for each gender when playing same vs. mixed-gender games is crucial to plan and organize the best training settings for this population to achieve broad health improvements. Nevertheless, one study limitation is the fact that during this study the participants were only evaluated during 4 TH training sessions (2 same- and 2 mixed-gender matches).

Given the practical value of organising mixed-gender games-based exercise interventions, future randomized controlled studies aiming at assessing participants’ health impact are needed.

## Conclusion

Cardiovascular demands were higher for middle-aged and elderly women than for age-matched men during mixed-gender matches. In men, same-gender game formats were more demanding than mixed-gender game formats, while in women the inverse occurred. Interestingly, fun levels were reported as being very high for both genders, independently of the gender game format, which can possibly lead to greater long-term adherence to this exercise program.

It could be concluded that recreational TH is an intermittent high-intensity and motivating exercise mode with potential to induce several health improvements for middle-aged and elderly men as well as for women, regardless the gender game format. From a practical point of view, same- and mixed-gender matches may be organised with the aim to promote health and physical fitness for this population. Since women are more physically and physiologically challenged when playing with men, a lead-in period with same-gender formats may be recommended for women, when implementing mixed-gender recreational TH-based exercise interventions.
